# Uncorking the limitation—improving dual tasking using transcranial electrical stimulation and task training in the elderly: a systematic review

**DOI:** 10.3389/fnagi.2024.1267307

**Published:** 2024-04-08

**Authors:** Yong Jiang, Perianen Ramasawmy, Andrea Antal

**Affiliations:** Department of Neurology, University Medical Center, Georg August University of Göttingen, Göttingen, Germany

**Keywords:** non-invasive brain stimulation, multitasking, task training, elderly, tDCS

## Abstract

**Introduction:**

With aging, dual task (DT) ability declines and is more cognitively demanding than single tasks. Rapidly declining DT performance is regarded as a predictor of neurodegenerative disease. Task training and non-invasive transcranial electrical stimulation (tES) are methods applied to optimize the DT ability of the elderly.

**Methods:**

A systematic search was carried out in the PUBMED, TDCS (transcranial direct current stimulation) databases, as well as Web of Science, and a qualitative analysis was conducted in 56 included studies. Aiming to summarize the results of studies that implemented tES, task training, or the combination for improving DT ability and related performance changes in healthy elderly and geriatric patients. For different approaches, the training procedures, parameters, as well as outcomes were discussed.

**Results:**

Task training, particularly cognitive-motor DT training, has more notable effects on improving DT performance in the elderly when compared to the neuromodulation method.

**Discussion:**

Anodal transcranial direct current stimulation (tDCS) over the left dorsolateral prefrontal cortex (L-DLPFC), or its combination with task training could be promising tools. However, additional evidence is required from aged healthy people and patients, as well as further exploration of electrode montage.

## Introduction

Walking while answering the phone, talking while preparing a meal, or texting on the bus while maintaining a standing balance, dual task (DT) happens frequently in day-to-day life. Compared with performing one task in isolation, carrying out concurrently two tasks can deteriorate mutual performance, as the cognitive system has a limited capacity for attending to several attention-demanding channels simultaneously (Navon and Gopher, 1979).

Potential factors affecting DT capacity in old adults were discussed in previous studies. It has been identified that attention as well as execution factors are the most critical predictors of DT performance, and aging-induced decline in these factors is closely related to the increased risk of falls and impaired cognition in the elderly (Holtzer et al., 2005; Ali et al., 2013; Harada et al., 2013; Sugata et al., 2018). Besides, previous study reported larger impairment in DT performance was observed in dementia patients when compared to single task (ST) performance, suggesting that DT deficit is a highly specific and sensitive indicator of cognitive decline, and can be regarded as a significant predictor of neurodegenerative diseases including Parkinson’s disease (PD) and Alzheimer’s disease (Sala and Logie, 2001; Schwenk et al., 2010; Montero-Odasso et al., 2017; Raichlen et al., 2020). The prefrontal cortex, which is associated with cognitive and motor function, has demonstrated greater activation during DT gait/balance in the healthy elderly when compared with healthy young adults (Kahya et al., 2019). The dorsolateral prefrontal cortex (DLPFC) plays a key role in cognition and executive control functions, e.g., working memory, inhibition, and task switching (Badre and Wagner, 2004; Brzezicka et al., 2019; Hertrich et al., 2021).

To optimize the DT capacity, different approaches were proposed, which can be summarized into two categories: (1) neuromodulation by applying low-intensity transcranial electrical stimulation (tES) and (2) skills acquisition based on task training.

TES is a safe neurophysiological method, which can regulate cortical excitability by altering the membrane potential and neural synchronization in a non-invasive manner (Bikson et al., 2016; Miyaguchi et al., 2020). It mainly includes transcranial direct current stimulation (tDCS) and transcranial alternating current stimulation (tACS). The former can modulate the membrane potential of neurons and therefore alter the spontaneous firing rates of neurons (Antal and Paulus, 2008). Anodal tDCS over the primary motor cortex (M1) tends to increase cortical excitability, while cathodal tDCS over M1 tends to decrease the excitability (Nitsche and Paulus, 2000). However, the net consequences of the stimulation depend on multiple factors including the state of the brain, the number of stimulation sessions, the stimulation duration, and the stimulation intensity (Bikson et al., 2019). Previous studies demonstrated that 20 min of anodal tDCS over the M1 at 2 mA reduced lower back pain (Jiang et al., 2020), while tDCS with the same parameters over the left DLPFC (L-DLPFC) enhanced working memory in old adults (Satorres et al., 2022). On the other hand, TACS modulates brain oscillations and is primarily used for improving cognitive function, and the effect is mainly affected by the frequency (Antal et al., 2008; Antal and Paulus, 2013). TACS over the DLPFC delivered at a gamma frequency has been shown to enhances working memory in healthy adults (Hoy et al., 2015) and executive function in mild cognitive impairment (MCI) patients (Kim et al., 2021).

The effectiveness of physical training in improving walking ability and balance has also been demonstrated in the elderly (Varela-Vasquez et al., 2020). A longitudinal neuroimaging study with a mean follow-up of 4 years found a close relationship between the volume loss of brain and memory, verbal fluency, visuospatial, as well as attention decline (Armstrong et al., 2020). On the other side, it has been reported that after a 6-month aerobic training, an increase in brain volume, gray matter, and white matter was observed in healthy elderly participants (Colcombe et al., 2006), whereas MCI patients showed improved cognitive function after a 12-week training (Alfini et al., 2019). Another study implementing a 3-month dual aerobic-cognitive training in healthy elderly adults found improved executive function and working memory when compared with the performance of pure cognitive training and pure aerobic training groups. The study conducted by Nouchi et al. (2021) demonstrated that attention, working memory, and processing speed can be facilitated by cognitive training. Faraza et al. (2021) observed that improved cognitive ability after a 4-week neuropsychological training could be attributed to higher functional connectivity within the DLPFC. Such evidence provides a basis for the argument that task training, including both ST and DT training, can be used for promoting DT performance (De Freitas et al., 2020; Chiaramonte et al., 2022). However, only few reviews discussed its effects in old participants (Gallou-Guyot et al., 2020; Khan et al., 2022). Besides, it has been hypothesized that the combination of these two methods may provide greater efficacy and feasibility for DT improvement in the elderly.

To our knowledge, this review is the first one to assess the relationship between potential therapeutic approaches such as tES and task training and DT performance in the elderly. We aimed to provide an overview of the previously implemented methods for enhancing DT performance in the healthy elderly and geriatric patients. Our objective was also to provide a reference for future home-based training as well as clinical rehabilitation of DT performance in the elderly. For studies that applied tES, the intensity, electrode montage, and stimulation duration were discussed, while for the training studies, we focused on the training tasks, training procedures, and the number of training sessions. Moreover, this review aimed to summarize the results of tES and training, when delivered either in combination or on its own.

## Methods

This systematic review was reported according to the Preferred Reporting Items for Systematic Reviews and Meta-Analyses (PRISMA) statement (Page et al., 2021).

### Databases and keyword searches

A literature database search for published studies including tES and training, both alone and in combination was conducted in PubMed (U.S. National Library of Medicine), tDCS database (Grossman et al., 2018) and Web of Science from their inception until December 2023. The database search was first done in PubMed, followed by the tDCS database and Web of Science. We screened all abstracts for relevance. For the tES-related articles, the search phrase was: [(tDCS) OR (Transcranial direct current stimulation) OR (tACS) OR (Transcranial alternating current stimulation) OR (noninvasive) OR (brain stimulation)] AND [(dual task) OR (multitasking) OR (dual cost)]. For the training-related articles, the search phrase was as follows: (training) AND [(dual task) OR (cognitive-motor) OR (simultaneous) OR (cognitive-physical) OR (multitasking)]. The same keywords were used for both databases.

### Selection criteria

Studies that met the following criteria were included: (1) healthy elderly, or geriatric patients diagnosed with neurological conditions such as stroke and dementia or musculoskeletal diseases such as osteoarthritis, (2) tES or single/dual task training applied either on their own or in combination, (3) single or multiple intervention sessions were performed, and (4) DT performance was assessed before and after the intervention. Studies were excluded if they were published in languages other than English. Studies were excluded if they: (1) were protocol papers, (2) were letters, (3) participants were children or the mean age <60 years old, (4) participants were individuals with partial loss of limbs, (5) primary outcomes were not related to DT ability, (6) were review or meta-analysis papers, (7) were case reports, (8) were commentary papers, and (9) were conference presentations.

### Risk of bias assessment

The authors (YJ and PR) assessed the risk of bias via the bias assessment tool—Cochrane risk-of-bias tool, independently. Three grades were assigned to each study individually: Low risk of bias, Some concerns, or High risk of bias. “Low risk of bias” was given when the study effectively addressed risks and elucidated its study design. Conversely, the “Some concerns” classification was given if a study failed to adequately specify details, leaving uncertainties about potential risks. A “high risk” was assigned if a study exhibited serious risks that could significantly impact outcomes due to a biased study design.

## Results

### Study characteristics

**FIGURE 1:**
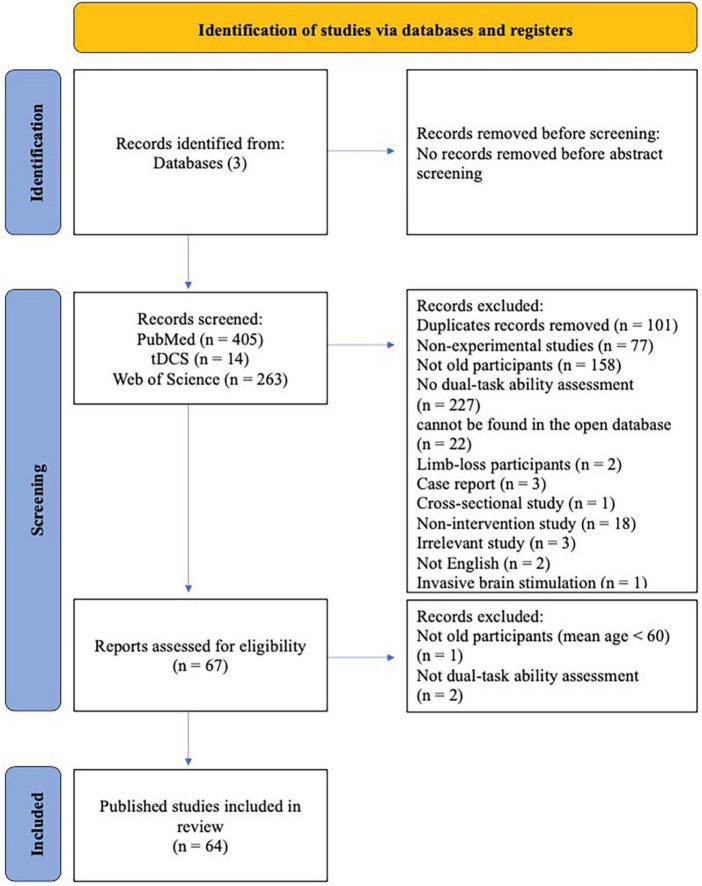
PRISMA flow diagram illustrating identification, screening, and inclusion strategies for the selection of articles.

**FIGURE 2:**
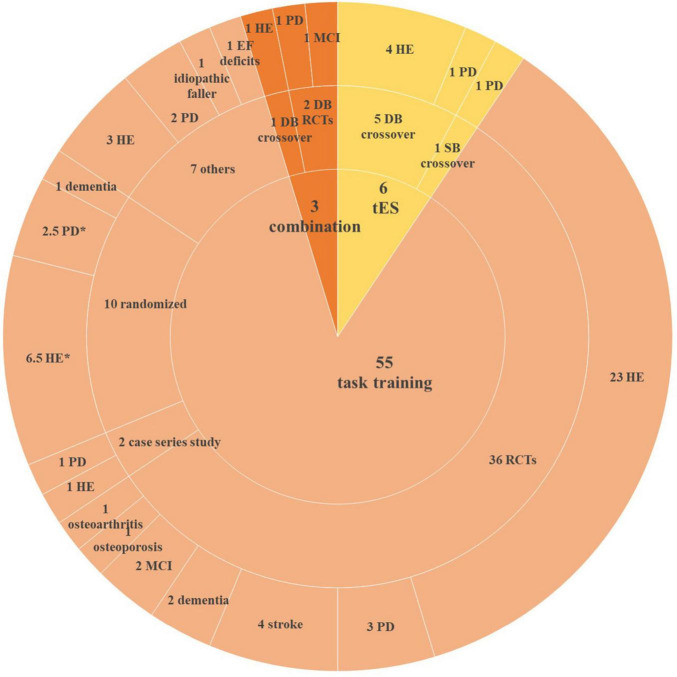
Characteristics of included studies. HE, healthy elderly; PD, Parkinson’s disease; EF, executive function; DB, double-blinded; SB, single-blinded; RCT, randomized clinical trial; MCI, mild cognitive impairment. *One study not only recruited healthy participants and PD patients but also assigned them to the same group.

Out of 682 articles screened, 64 studies published between 2005 and 2023 were included in this review (Figure 1). Study characteristics are shown in Figure 2.

Our review included six tES studies (five applied tDCS and one applied both tDCS and tACS), of which five were double-blinded randomized controlled trials (RCTs) and one was a single-blinded RCT which recruited PD patients. Out of these five RCTs, four recruited healthy participants and one recruited PD patients.

Fifty-five studies applied only task training. Thirty-six studies were RCTs, of which 24 studies recruited healthy participants and 12 studies recruited geriatric patients, including PD, stroke, dementia, MCI, as well as osteoporosis and osteoarthritis patients. Two studies were case series studies, recruited healthy participants and PD patients separately. Ten studies were randomized studies (participants were assigned to different groups randomly), of which six studies allocated healthy elderly, two studies recruited PD patients, one with dementia patients, and the other one recruited both healthy and PD participants. Seven studies did not mention their designs.

Three studies combined anodal tDCS with task training. All of them were double-blinded RCTs, recruited healthy participants, MCI patients, and PD patients, respectively.

### Risk of bias assessment

**FIGURE 3:**
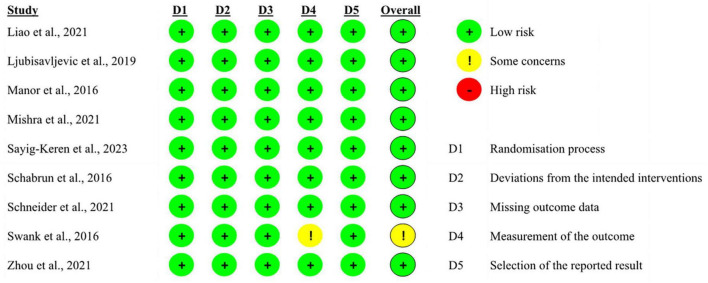
Risk of bias of included tES-related studies based on authors’ judgment.

**FIGURE 4:**
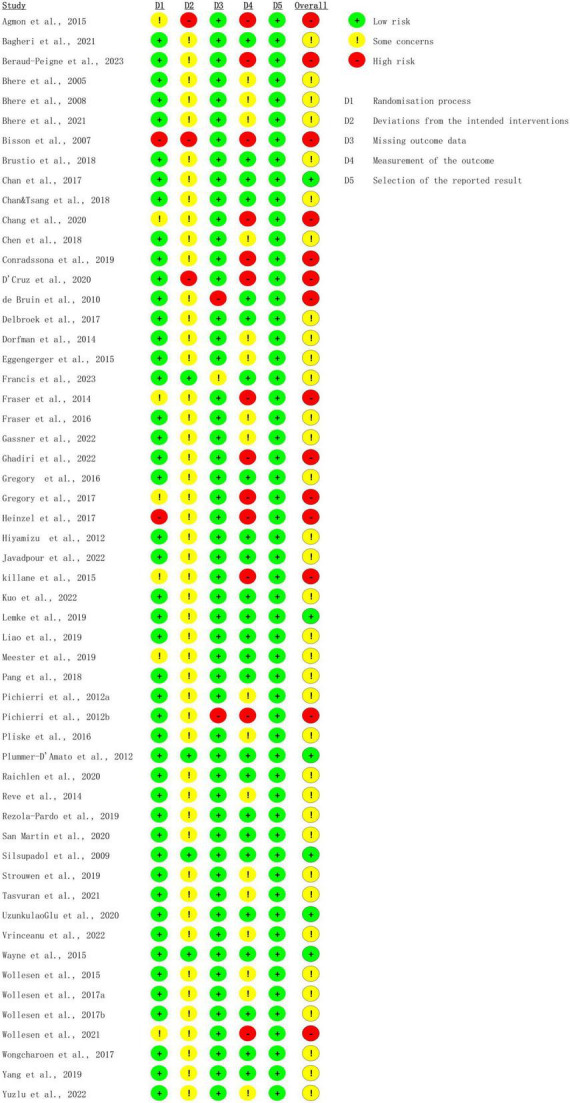
Risk of bias of included task training studies based on authors’ judgment.

**FIGURE 5:**
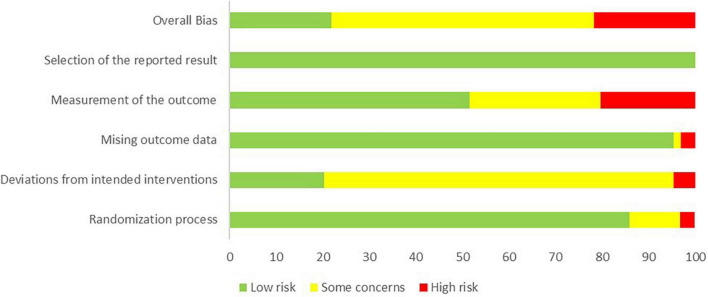
Risk of bias assessment based on subscales for all included studies based on authors’ judgment.

The overall bias in tES-related studies was low, only one study had a concerned risk in measurement (Figure 3). Thirteen training studies had high risk in measurement and 48 studies had concerned risk in intended intervention (Figure 4), as the training conductor might also participated in the data analysis in these studies (Figure 5). Nevertheless, this phenomenon is explicable and understandable. Compared with tES-related studies, which employ shorter durations (20–30 min) and fewer sessions (mostly only 1), the majority of training studies implemented an intervention of 60 min with more than 12 sessions.

### Intervention characteristics

#### tES only intervention

All six studies applied a crossover design, whereby all participants received both anodal and sham stimulation with a different washout period, ranging from 3 days to 2 weeks. Four of them applied a single session of classical anodal tDCS intervention at a 1.5–2 mA over the L-DLPFC for a duration ranging from 20 to 30 min (Table 1), while one study applied a single session of 4 mA, 20-min high-definition tDCS (HD-tDCS) (Zhou et al., 2021). The other one applied both anodal-tDCS and tACS over L-DLPFC and the left frontoparietal network (L-FNP) separately for 20 min with the current at 1.5 mA (Sayig-Keren et al., 2023).

**TABLE 1:** Summary of tDCS parameters used to enhance DT in old adults and task performance.

References	Study design	Participants (*N*, female/ male)	Age (mean ± SD)	Stimulation protocol									Task	Outcome	
				Polarity	Electrodes size (cm)	Target area	Montage (anodal and cathodal)		Intensity (mA)	Duration (min)	Ramp-up (s)	Intervention sessions		DT performance	ST performance
Ljubisavljevic et al., 2019	Double-blinded, randomized, crossover, sham-controlled	HE *N* = 22, 6/16	62.6 ± 3.2	atDCS	5 × 7	L-DLPFC	F3	FP2	1.5	30	30	1 (visit: 1 real, 1 sham)	GPT, SSST, SRT Dual-task: GPT + SSST	No significant change	No significant change
						R-DLPFC	F4	FP1						No significant change	
						Bilateral L-DLPFC	F3	F4						DTC to SSST ↓* during stimulation	
						Bilateral R-DLPFC	F4	F3						No significant change	
				Sham		Randomly			1.5	1	30			No significant change	
Manor et al., 2016	Double-blinded, crossover, sham-controlled	HE *N* = 37, 25/12	61 ± 5	atDCS	5 × 7	L-DLPFC	F3	FP2	2	20	30	1 (visit: 1 real, 1 sham)	Walking (50 m), standing (60 s), SSST Dual task: walking/ standing + SSST	DTC to standing sway area, standing sway velocity, walking speed, subtraction error rate ↓*	No significant change
				Sham						1	30			No significant change	
Mishra and Thrasher, 2021	Double-blinded, crossover, sham-controlled	PD patients *N* = 20, 6/14	67.8 ± 8.3	atDCS	5 × 7	L-DLPFC	F3	FP2	2	30	/	1 (visit: 1 real, 1 sham)	Walking (30 m), PVF Dual task: walking + PVF	Gait speed ↑* immediately and 15 min after; PVF ↑* 15 and 30 min later compared to sham; DTC to gait speed ↓* 30 min after compared to sham	Gait speed ↑* 15 min after; PVF score ↑* during and after
				Sham						1				No significant change	Gait speed ↑* after stimulation; PVF score ↑* during and after
Sayig-Keren et al., 2023	Double-blinded, randomized, crossover, sham-controlled	HE *N* = 20, 9/11	72.6 ± 5.0	atDCS	π	L-DLPFC	F3	AF4, FC1, FC5	1.5	20	/	1 (visit: 1 tD, 1 tA, 1 sham)	Walking (25 m), SCWT, SDMT, DSMT Dual task: walking + subtraction	DTC to step length ↓*	No significant change
				6 HZ tACS		Left fronto-parietal network (L-FPN)	F3, P3	Cz	3 (peak–peak)					DTC to gait speed ↓*	
				Sham		Left fronto-parietal network (L-FPN)	F3, P3	Cz	1.5	0.5				No significant change	
Swank et al., 2016	Single-blinded, randomized, sham-controlled, crossover	Elderly with PD *N* = 10, 2/8	68.7 ± 10.2	atDCS	π	DLPFC	F3	F4	2	20	30	1 (visit: 1 real, 1 sham)	TUG, subtraction task Dual task: TUG + water carrying/ subtraction task	No significant change, but a trend toward a reduction in the DTC to center-of-pressure	No significant change
				Sham					1	0.5				No significant change	
Zhou et al., 2021	Double-blinded, randomized, sham-controlled, crossover	HE *N* = 57, 43/14	75 ± 5	HD-tDCS	π	L-DLPFC + SM1	F3, Cz	AF4, CP1, FC1, FC5	Single electrode ≤1.5; total current <4	20	59	1 (visit 4 times with washout period)	Standing (30 s), walking (20 m), SCWT, DSST Dual-task: standing + subtraction task; walking + subtraction task	Sway speed/sway area ↓* in DT standing; DTC to standing sway speed, standing sway area, walking speed ↓*	No significant change
						L-DLPFC	F3	AF4, FC1, CP1						Sway speed/sway area ↓* in DT standing; DTC to postural sway speed, standing sway area, walking speed ↓*	
						SM1	Cz	AF4, FC1, FC5						No significant change	
				Acti-sham		L-DLPFC + SM1	Cz, FC1	F3, CP1	0.25 mA at Cz and FC1					No significant change	

a, anodal; HE, healthy old adults; PD, Parkinson’s disease; FOG, freezing of gait; SCWT, Stroop Color and Word Test; DSST, Digit Symbol Substitution Test; GPT, Grooved Pegboard Test; SSST, Serial Seven Subtractions; SRT, simple reaction time; SDWT, Symbol Digit Modalities Test; DSMT, Digit Span Memory Test; PVF, phoneme verbal fluency task; TUG, Timed Up and Go. The symbol “/” represents no report.

*Indicates significant.

#### Training-only intervention

Fifty-five studies implemented only task training, with a maximum of 78 sessions. The intervention programs included: (1) single motor task training (balance standing, walking, or aerobic training), (2) single cognitive task training (visual or auditory working memory task training), (3) dual cognitive task training (visual and auditory working memory task training), (4) dual motor task training (walking while bouncing a basketball), and (5) cognitive-motor dual task training (walking while performing visual recognition task) (Tables 2, 3). Participants in the control group were normally asked to keep their daily activities or perform simple ST, for example, walking at a self-suitable speed.

**TABLE 2:** Summary of the training procedures used to enhance DT in healthy elderly and task performance.

References	Study design	Group	Participants (*N*, female/ male)	Age (mean ± SD)	Training task	Sessions duration (min), frequency	Assessment timepoint	Assessment tasks	Outcome	
									DT performance	ST performance
Agmon et al., 2015	/	Fitness training	HE *N* = 28, 25/3	74.5 ± 7.9	Cardiovascular endurance, strength training, stretching, balance and posture training	18 60 min, 2–3/week	Pretraining and post training	TUG, backward counting, 1-min walk, verbal fluency, BBS, Mini-balance evaluation system test (Mini-BEST), FSST, TMT-b, SCWT; DT: TUG + backward counting/glass of water carrying, 1-min walking + verbal fluency	DTC to TUG-counting ↑*, DTC to walking-verbal ↑*; TUG, 1-min walking ↑*	TUG, 1-min walk, BBS, Mini-BEST, FSST, TMT-B, SCWT ↑*
Bagheri et al., 2021	Single-blinded, randomized, controlled	Video-game group	HE *N* = 31, 21/10	71.83 ± 4.24	Video games in Nintendo Wii. Static balance game: single leg extension, Torso Twist; dynamic balance game: Table Tilt, Penguin Fishing, Soccer Heading, Tightrope Walk	16 60 min, 3/week	Pretraining, post training, and 8-week follow-up	Standing still (30 s); DT: standing + Digit Memory Test	DTC of SD of velocity (anteroposterior)/the Lyapunov function (anteroposterior and mediolateral) ↓* after and follow-up	/
		Cognitive-motor DT group with VP	HE *N* = 29, 18/11	71.22 ± 5.82	Standing/ walking/ stepping/ reaching/ sit to stand/ball kicking/ball throwing and catching + verbal fluency/backward counting task					
Beraud-Peigne et al., 2023	Randomized	Gaming group	HE *N* = 19, /	69.63 ± 5.31	Immersive and Interactive Wall Exergames	24 60 min, 2/week	Pretraining and post training	ST: the Zoo Map Test, The Spatial Span Test, The Stroop Test, The Mental Rotation Test, TMT, The physical battery, TUG test DT: single recall test + TUG	DT time ↓*	The Spatial Span Test, Stroop test ↑*
		Active control group	HE *N* = 15, /	70.27 ± 5.82	Muscle-strengthening exercises				No significant change	No significant change
Bherer et al., 2005	Randomized, controlled	VP group	HE *N* = 36, 17/19	70 ± 7	Single task: auditory discrimination task; visual identification task Dual task: auditory + visual identification task	5 60 min, within 3 weeks	Pretraining and post training	Dual task: auditory discrimination task (involves a new set of sounds) + visual identification task (involves new numbers); double visual identification tasks	DTC to reaction time ↓* after dual-task training	Response accuracy ↑*
		FP group							Response accuracy of dual-task ↑* after dual-task training No main effect of training condition (VP vs. FP)	
		Control group			No training				No significant change	No significant change
Bherer et al., 2008	Randomized, controlled	VP group	HE *N* = 18, 24/20	70.38 ± 5.9	Letter discrimination task, color discrimination task DT: letter + color discrimination	5 60 min, within 3 weeks	Pretraining and post training	DT: number + pattern discrimination, tone + letter discrimination, tone + number discrimination	DTC to reaction time ↓* after training	Response accuracy ↑* after task training
		FP group	HE *N* = 14, /						DTC to reaction time ↓* after training	Response accuracy ↑* after task training
		Control group	HE *N* = 12, /	71.67 ± 7.0	No training				No significant change	No significant change
Bherer et al., 2021	Randomized	Cognitive-motor DT group	HE *N* = 26, 17/9	71.85 ± 7.16	Visual dual discrimination task + aerobic training	36 60 min, cognitive tasks: 2/week, aerobic tasks: 1/week	Pretraining and post training	Dual visual discrimination task (new)	DTC to accuracy ↑* after training	/
		Cognitive-motor active control group	HE *N* = 23, 19/4	74.17 ± 6.71	Visual dual discrimination task + stretching exercise				DTC to reaction time ↓* after training; DTC to accuracy ↑*	
		Cognitive active control-motor group	HE *N* = 20, 11/9	70.75 ± 6.90	Computer lesson + aerobic training				No significant change	
		Cognitive active control-motor active control group	HE *N* = 18, 15/3	72.50 ± 6.96	Computer lesson + stretch training				No significant change	
Bisson et al., 2007	/	VR DT group	HE *N* = 12, 5/7	74.4 ± 3.65	Dynamic balance training with VR	20 30 min, 2/week	Pretraining, post training, and 4-week follow-up	Standing (60 s), community balance and mobility scale (CB&M) DT: standing + tone discrimination	RT ↓*	CB&M ↑*
		Biofeedback DT group	HE *N* = 12, 9/3	74.4 ± 4.92	Dynamic balance training with visual feedback				RT ↓*	CB&M ↑*
Brustio et al., 2018	Single-blinded, randomized, controlled	DT group	HE *N* = 19, 14/5	74.3 ± 2.6	DT: balance training + daily activities (unscrewing/screwing the bolt, making a knot…), walking + daily activities (put on/take off sweater, buttoning/unbuttoning a shirt…)	32 60 min, 2/week	Pretraining and post training	6-MWT, TUG, four-square step test (FFST); DT: 6-MWT/TUG/FFST + carrying a glass of water/ball	TUG, FFST ↑*	TUG, FFST ↑*
		ST group	HE *N* = 19, 14/5	75.2 ± 3.4	Balance training: semi-tandem standing, tandem standing, single leg standing Gait training: walking				/	
		Control group	HE *N* = 22, 14/8	74 ± 3.2	No training					
D’Cruz et al., 2020	Randomized	Tied-belt group	HE + PD patients *N* = 18, 3/15	67.38 ± 10.1	Walking training (both belts were set to the training speed)	1 30 min	Pretraining, post training, and 1-day after	DT: walking/turning in place (1 min) + auditory Stroop	Gait speed, stride length ↑* post	/
		SB75 group; steady speed ratio 0.75:1	HE + PD patients *N* = 20, 7/13	68.1 ± 10.0	Walking training (one split belt in training speed while one in 75% speed)				Gait speed, stride length ↑* post; Stroop response time, accuracy ↑*; peak turning speed ↑* compared with TB group	
		SB50 group; steady speed ratio 0.5:1	HE + PD patients *N* = 21, 8/13	69.33 ± 7.22	Walking training (one split belt in training speed while one in 50% speed)				Gait speed, stride length ↑* post and after, step width variability ↓* post; Stroop response time, accuracy ↑*; peak turning speed ↑* compared with TB group	
		SBCR group: changing speed between 0.75:1 and 0.5:1	HE + PD patients *N* = 22, 9/13	71.09 ± 7.60	Walking training (one split belt in training speed while one alternated between 75% and 50% speed)				Gait speed, stride length ↑* post and after; Stroop response time, accuracy ↑*; peak turning speed ↑* compared with TB group	
Delbroek et al., 2017	Randomized, controlled	VR DT group	HE *N* = 10, 8/2	86.9 ± 5.6	DT training using Bio Rescue	12 Training time increased gradually, 18–30 min, 2/week	Pretraining and post training	TUG, MoCA DT: TUG + visual task	No significant change	TUG score ↑*
		Control group	HE *N* = 10, 5/5	87.5 ± 6.6	No training				No significant change	
Eggenberger et al., 2015	Randomized, controlled	VR video game dancing group	HE *N* = 24, 14/10	77.3 ± 6.3	Video game dancing, strength, and balance training	52 60 min, 2/week	Pretraining, during (3 months), post training, and 1 year follow-up	Walking, SPPB, 6-MWT DT: walking + counting/enumerating task	DTC of step time variability ↓*	Step time ↓*, SPPB, 6-MWT ↑* during and after
		Walking with memory training group	HE *N* = 22, 16/6	78.5 ± 5.1	DT: treadmill walking + verbal WM, strength, and balance training				DTC of step time variability, DTC to gait variability ↓*;	SPPB, 6-MWT ↑* during and after
		Treadmill walking group	HE *N* = 25, 16/9	80.8 ± 4.7	Treadmill walking				No significant change	SPPB, 6-MWT ↑* during and after
Fraser et al., 2016	Randomized	Motor-cognitive training group	HE *N* = 21, /	71.90 ± 6.84	Resistance/cardiovascular training + visual discrimination task	36 60 min, 3/week	Pretraining and post training	N-back, walking (30 s), standing (20 s); DT: walking/standing + n-back	Gait speed ↑*	Gait speed, accuracy ↑*
		Motor-computer lesson group	HE *N* = 17, /	70.53 ± 7.34	Resistance/cardiovascular training + computer lessons					
		Stretch-cognitive training group	HE *N* = 18, /	72.22 ± 5.93	Whole body stretch exercise + visual discrimination task					
		Stretch-computer lesson group	HE *N* = 16, /	71.13 ± 5.40	Whole body stretch exercise + computer lessons					
Gregory et al., 2016	Single-blinded, randomized, controlled	Exercise DT group	HE *N* = 23, 15/8	72.6 ± 7.4	ST: aerobic training DT: square-stepping exercise + verbal fluency task/arithmetic task	48–72 60–75 min, 2–3/week	Pretraining, during (12 week), post training, and 26-week follow-up	Walking DT: walking + naming task/subtraction task	Gait speed, step length ↑*; stride time variability ↓*	No significant change
		Exercise group	HE *N* = 21, 15/6	74.5 ± 7.0	Aerobic training, square-stepping exercise				No significant change	
Gregory et al., 2017	Case series study	DT group	HE *N* = 56, 34/22	70.4 ± 6.2	Aerobic training, walking + verbal fluency/arithmetic	78 40 min, 3/week	Pretraining, during, post training, and 26-week follow-up	TMT, digit symbol coding (DSC), verbal fluency task, auditory verbal learning test (AVLT), walking DT: walking + subtraction task	Step length, speed ↑* post	TMT time ↓* after and follow-up DSC, phonemic verbal fluency ↑* post and follow-up; step length, speed ↑* post
Heinzel et al., 2017	/	Working memory training group	HE *N* = 18, 11/7	65.78 ± 3.04	Visual/auditory working memory (0, 1,2, 3) task DT: visual task + auditory task	12 45 min, 3/week	Pretraining, during, and post training	Visual/auditory working memory task (load 1, load 2) DT: visual WM + auditory WM task	Accuracy ↑*, DTC to working memory ↓* during, DTC to load 1 ↓* after training	Visual WM ↑*
		Control group	HE *N* = 16, 11/5	65 ± 3.67	No training				No significant change	No significant change
Hiyamizu et al., 2012	Single-blinded, randomized, controlled	DT group	HE *N* = 17, 10/7	72.9 ± 5.1	DT: strength/balance + cognitive (calculation, visual search, verbal fluency task) training	24 60 min, 2/week	Pretraining and post training	Chair Stand Test (CST), Functional Reach Test (FRT), TUG, TMT DT: standing (30 s) + Stroop task	No significant change	Stroop task ↑*
		ST group	HE *N* = 19, 16/3	71.2 ± 4.4	Strength/balance training					No significant change
Javadpour et al., 2022	Randomized, controlled	ST balance training group	HE *N* = 23, 16/7	67.65 ± 2.42	Balance training: narrow-base standing, tandem standing, walking	18 40–60 min, 3/week	Pretraining and post training	Walking, TUG, Fullerton Advanced Balance (FAB) Scale, Actives-specific Balance Confidence (ABC) Scale DT: walking + backward counting	Gait speed ↑*	Gait speed, FAB, ABC ↑*
		DT balance training group	HE *N* = 23, 18/5	68.86 ± 3.48	DT: balance training + naming task/backward counting				Gait speed ↑*	Gait speed, FAB, ABC ↑*
		Control group	HE *N* = 23, 15/8	69.34 ± 3.77	No training				/	/
Pichierri et al., 2012a	Randomized, controlled	DT group	HE *N* = 9, 6/3	83.6 ± 3.4	Resistance training, balance, dance video games	24 60 min, 2/week	Pretraining and post training	Voluntary step execution DT: voluntary step execution + SCWT	Initiation time of forward/backward steps ↑*	/
		Control group	HE *N* = 6, 3/3	86.2 ± 4.8	Daily activity of care homes				No significant change	
Pichierri et al., 2012b	Randomized, controlled	Dance group	HE *N* = 11, 8/3	86.9 ± 5.1	Strength, balance training, video dance gaming	24 50–55 min, 2/week	Pretraining and post training	Normal walking, fast walking DT: normal/fast walking + subtraction task	DTC to gait speed, step length, double support time ↓* in fast walking + subtraction; gait speed, cadence ↑*, step time, cycle time, stance time, single/double support time ↓* in DT	Cadence ↑*, step time, cycle time, stance time, single support time ↓* in normal walking; cadence ↑*, step time, stance time ↓* in fast waling
		Control group	HE *N* = 11, 10/1	85.6 ± 4.6	Strength, balance training	24 40 min, 2/week			DTC to single support time in fast/normal walking + subtraction ↑*; gait speed ↑* in normal walking + subtraction task; gait speed ↑*, single support time ↓* in fast walking + subtraction	Cadence ↑*, step time, cycle time, stance time, single/double support time ↓* in normal walking
Pliske et al., 2016	Randomized, controlled	Karate training group	HE *N* = 25, 14/11	69.92 ± 4.1	Karate	20 60 min, 2/week	Pretraining and post training	Walking Cognitive DT (CDT): walking + subtraction task Motor DT (MDT): walking + carrying a glass of water	CDT: calculation speed, gait speed, cadence ↑*, step time ↓* MDT: gait speed, cadence, step length ↑*	Gait speed, cadence, step length ↑*, step time ↓*
		Fitness group training	HE *N* = 24, 16/8	68.71 ± 4.9	Balance, strength, coordination training				CDT: calculation speed in CDT, gait speed, cadence, step length ↑* MDT: gait speed, cadence ↑*	Gait speed, cadence, step length ↑*, step time ↓*
		Control group	HE *N* = 19, 11/8	68.74 ± 4.3	No training				CDT: accuracy, calculation speed, gait speed, step length ↑* MDT: cadence, gait speed, step length ↑*, step time ↓*	Gait speed, step length ↑*, step time ↓*
Plummer-D’Amato et al., 2012	Randomized, controlled	ST group	HE *N* = 7, 7/0	76.7 ± 6.0	Balance: standing, walking; gait: obstacle negotiation tasks; agility: rope ladder	4 45 min, 1/week	Pretraining and post training	6-min walking, Timed 25-Foot Walk, TUG DT: walking + spontaneous speech/alphabet recitation/coin transfer	No significant change	TUG, gait speed ↑*
		DT group	HE *N* = 10, 9/1	76.6 ± 5.6	Balance/gait/agility training + random number generation/ Word association/backward recitation/working memory					
Raichlen et al., 2020	Single-blinded, randomized, controlled	Cognitive training group	HE *N* = 21, 14/7	66.35 ± 3.89	Verbal paired-association, Simon inhibition task, letter switching, N-back, simple and choice reaction time, verbal paired-associates memory condition	36 30 min, 3/week	Pretraining, during (6-week), and post training	Walking DT: walking + subtraction task	Accuracy ↑* after training	No significant change
		Motor training group	HE *N* = 19, 11/8	68.1 ± 3.92	Bicycle riding				Accuracy ↑* after training	
		Motor-cognitive DT group	HE *N* = 20, 13/7	67.67 ± 4.65	Combination of above				Accuracy ↑* during and after training	
		Control group	HE *N* = 14, 9/5	69.28 ± 4.34	Video watching				No significant change	
Reve and de Bruin, 2014	Randomized, controlled	Strength-balance (SB) training group	HE *N* = 82, 52/30	81.9 ± 6.3	Strength training, balance training	24 30 min, 2/week	Pretraining and post training	Normal/fast walking, TMT, short physical performance battery (SPPB), expanded timed get-up-and-go (ETGUG) test, simple reaction time task DT: normal walking + subtraction task/naming task, fast walking + enumerating	DTC to gait speed, step time, step length ↓* in walking;	SPPB, TMT ↑*; RT ↓*,
		SB-cognitive training group	HE *N* = 74, 49/25	81.1 ± 8.3	Strength training, balance training, cognitive training (alert/divide training task, 10 min, 3/week)				DTC to gait speed, step time, step length ↓* in walking;	SPPB, DTGUG, TMT ↑*; RT ↓*
Rezola-Pardo et al., 2019	Randomized, controlled	Multicomponent group	HE *N* = 43, 28/15	85.3 ± 7.1	Strength, balance training	24 60 min, 2/week	Pretraining and post training	SPPB, Senior Fitness Test (SFT), TUG, MoCA, Symbol Search and Coding tests, semantic fluency test, verbal fluency test, Rey Auditory Verbal Learning Test (RAVLT), TMT-a DT: walking + backward counting/naming animals/Go-on-Go test	Gait speed ↑*	Gait speed, SFT, SPPB, TUG ↑*
		DT group	HE *N* = 42, 29/13	84.9 ± 6	DT: strength/balance + cognitive training				Gait speed ↑*	Gait speed, SFT, SPPB ↑*
Silsupadol et al., 2009	Double-blinded, randomized, controlled	ST balance training group	HE *N* = 7, 7/0	74.71 ± 7.80	Standing, walking	12 45 min, 3/week	Pretraining and post training	walking, BBS DT: walking + mathematic task	No significant change	BBS ↑*
		Cognitive-motor DT training with FP	HE *N* = 8, 6/2	74.38 ± 6.16	DT: standing/walking + objects naming/number remembering				No significant change	
		Cognitive-motor DT training with VP	HE *N* = 6, 4/2	76.01 ± 4.65	Gait speed ↑*					
Tasvuran et al., 2021	Randomized, controlled	ST group	HE *N* = 16, 9/7	64.6 ± 3.3	Standing, single leg standing, walking, reaching	12 60 min, 2/week	Pretraining and post training	10-min walk test., standardized mini-mental state exam (SMMSE), SCWT DT: training tasks	No significant change	Gait speed, cadence, step length ↑*
		DT group	HE *N* = 16, 8/8	65.6 ± 2.6	Standing/single leg standing/walking/ reaching + numbers recalling/letters drawing/backward counting				Gait speed, cadence, step length ↑*	Gait speed, cadence ↑*; step length ↑* compared with ST group SMMSE, SCWT ↑*
Trombini-Souza et al., 2023	Randomized, double-blinded, controlled	DT group	HE *N* = 30, 26/4	67 ± 5	ST: walking, obstacle crossing, football DT: ST + subtraction/ spelling/remembering Only DTs were performed in the second half of the course	48 60 min, 2/week	Pretraining and post training	ST: Stroop test, standing, TMT DT: Stroop + standing	DT performance ↑*	Standing, walking, Stroop ↑*
		Control group	HE *N* = 30, 26/4	66 ± 4	ST and DT were performed alternately					
Vrinceanu et al., 2022	Randomized	Aerobic training group	HE *N* = 26, 19/7	69.28 ± 4.85	High-intensity interval training with a recumbent bicycle	36 60 min, 3/week	Pretraining and post training	ST: VO2 peak, 10-min walking DT: dual visual discrimination tasks	No significant change	/
		Gross motor ability group	HE *N* = 27, 20/7	70.21 ± 5.86	Treadmill walking, one leg standing, balance training, walking sideways				DTC, reaction time ↓*	
		Cognition group	HE *N* = 25, 12/13	70.46 ± 6.07	N-back, Stroop test				Reaction time ↓*	
Wayne et al., 2015	Randomized, controlled	Tai Chi training group	HE *N* = 31, 22/9	63.94 ± 8.02	Tai Chi training, usual healthcare	52 30 min, 2/week	Pretraining and post training	Walking DT: walking + subtraction task	Gait speed ↑*	/
		Control group	HE *N* = 29, 18/11	64.45 ± 7.42	Usual healthcare	/			Gait speed ↑*	
Wollesen et al., 2015	Randomized, controlled	DT group	HE *N* = 19, 12/7	72.7 ± 4.7	Daily activities (brisk walking, avoiding obstacles, side steps, turns) DT: ST + visual stimulus	12 60 min, 1/week	Pretraining and post training	30-s walking test DT: waling + visual-verbal Stroop test	DTC to gait line ↓*; step width ↓* and more than control group, step line ↑*	Step width ↓*, step line ↑*
		Control group	HE *N* = 19, 12/7		No training				Step width ↓*	Step width ↓*
Wollesen et al., 2017a	Randomized, controlled	DT group	HE *N* = 29, 22/7	70.7 ± 4.9	Balance training; task managing training DT: balance and coordination tasks + task managing training	12 60 min, 1/week	Pretraining and post training	Walking (30 s), Stroop test DT: walking + Stroop test	Step length ↑*, step width ↓* (better than ST group)	Step length, gait-line ↑*, step width ↓* (better than ST group)
		ST group	HE *N* = 23, 15/8	71.7 ± 4.9	Resistance training				Step length ↑*, step width ↓*	Step length, gait-line ↑*, step width ↓*
		Control group	HE *N* = 26, 19/7	71.7 ± 5.0	No training				No significant change	No significant change
Wollesen et al., 2017b	Single-blinded, randomized, controlled	DT balance training group	HE (FES-I < 20) *N* = 26, 16/10	72.2 ± 4.6	Walking DT: walking + attention distraction task/visio-spatial and executive task	12 60 min, 1/week	Pretraining and post training	30-s walking test DT: waling + visual-verbal Stroop test	Step length, gait line ↑*	No significant change
			HE (FES-I > 20) *N* = 30, 28/2	69.8 ± 5.7						
		Control group	HE (FES-I < 20) *N* = 19, 12/7	72.9 ± 4.4	No training				No significant change	
			HE (FES-I > 20) *N* = 20, 17/3	72.7 ± 5.3						
Wongcharoen et al., 2017	Randomized, single blind	Motor group	HE *N* = 15, /	73.53 ± 5.94	Balance training	12 60 min, 3/week	Pretraining and post training	ST: verbal fluency task, counting, 6-min walking, obstacle crossing DT: walking + verbal fluency task, obstacle crossing + counting	DT balance, walking, verbal response ↑*	Balance, walking, verbal response ↑*
		Cognitive group	HE *N* = 15, /	72.40 ± 6.30	Attention training, working memory training					
		Dual motor-cognitive group	HE *N* = 15, /	71.87 ± 4.57	Balance + cognitive training					
		Dual cognitive-cognitive group	HE *N* = 15, /	74.73 ± 5.97	Cognitive + cognitive training				No significant change	No significant change
Yuzlu et al., 2022	Randomized	Integrated DT group	HE *N* = 29, 24/5	82.9 ± 6.6	Balance training (standing, one-leg standing, walking) + cognitive training (memory task, verbal fluency task…)	16 60 min, 2/week	Pretraining and post training	ST: BBS, TUG, Tinetti Falls Efficacy Scale (TTFE), 10-min walking DT: 10-min walking + counting, TUG + trail making test	Dual TUG, Dual walking ↑*	BBS, TUG, TTFE ↑*
		Consecutive DT group	HE *N* = 29, 23/6	85.3 ± 7.2	Balance training, followed by cognitive training					

HE, healthy elderly; VP, participants were required to vary their response priorities between two tasks; FP, attention was shared equally between the tasks; FES-I, the Falls Efficacy Scale International; 6-MWT, 6-min walk test; TUG, timed-up-and-go; MoCA, Montreal Cognitive Assessment; BBS, Berg Balance Scale; SCWT, Stroop Color and Word Test; TMT, Trail Making Test; FSST, Four Square Step Test; RT, reaction times; SPPB, short physical performance battery; VR, virtual reality; IQR, interquartile range. The symbol “/” represents no report;

*p < 0.05.

**TABLE 3:** Summary of the training procedures used to enhance DT in geriatric patients and task performance.

References	Study design	Group	Participants (*N*, female/ male)	Age (mean ± SD)	Training task	Sessions duration (min), frequency	Assessment timepoint	Assessment tasks	Outcome	
									DT performance	ST performance
Chan and Tsang, 2017	Single-blinded, randomized, controlled	Tai Chi group	Stroke patients *N* = 9, 4/5	63.9 ± 6.1	Yang-style Tai Chi	24 60 min, 2/week	Pretraining, post training, and follow-up	ST: Stroop test, stepping down DT: Stroop test + stepping down No significant change	Stroop test ↑* in the follow-up	No significant change
		Conventional exercise group	Stroke patients *N* = 5, 2/3	63.2 ± 9.7	Walking, limbs mobilization, stretching, and muscle strengthening				No significant change	No significant change
		Control group	Stroke patients *N* = 9, 5/4	63.2 ± 6.0	No training				No significant change	No significant change
Chan and Tsang, 2018	Single blinded, randomized, controlled	Tai Chi group	Stroke patients *N* = 15, 6/9	63 ± 7	Modified Yang-style Tai Chi training	24 60 min, 2/week	Pretraining, post training, and follow-up	ST: auditory Stroop test, walking DT: walking + auditory Stroop test	No significant change	No significant change
		Conventional exercise group	Stroke patients *N* = 17, 7/10	62.7 ± 7.3	Joint mobilization, stretching, and strengthening exercises					
		Control group	Stroke patients *N* = 15, 7/8	62.7 ± 7.3	No training					
Chang et al., 2020	/	/	PD patients *N* = 13, 4/9	60.64 ± 5.32	Bicycle riding, calculation/spatial memory task/Stroop color-word task	16 60 min, 2/week	Pretraining, during, and post training	Walking, UPDRS, TUG, calculation, spatial memory, SCWT DT: walking + calculation/spatial memory/SCWT	Gait speed, step length ↑*, step time, double limb support time ↓*	Gait speed ↑*; step time, double limb support time, UPDRS-III ↓*
Chen and Pei, 2018	Randomized, controlled	Music DT group	Patients with dementia *N* = 15, 9/6	77.3 ± 9.4	DT: singing/instrument playing + walking forward/side-stepping	8 60 min, 1/week	Pretraining and post training	TMT(a), walking, TUG DT: walking + forward/backward digit recall	No significant change	TMT time ↓*
		Control group	Patients with dementia *N* = 13, 5/8	77.3 ± 10	Playing chess/cards, reading, writing, mathematic exercise, puzzles, games					No significant change
Conradsson and Halvarsson, 2019	Randomized, controlled	DT balance training group	Women with osteoporosis *N* = 43	76 ± 6	DT: balance training + counting task/motor task (carrying, manipulating objects)	36 45 min, 3/week	Pretraining and post training	Walking DT: walking + reciting alphabet	Gait speed, cadence, step width ↑*, step time, swing time, stance time ↓*	Gait speed, cadence ↑*, step time, swing time ↓*
		Balance training group			Balance training: waling, standing, leaning, reaching				No significant change	
		Control group	Women with osteoporosis *N* = 25	76 ± 5	No training				No significant change	No significant change
de Bruin et al., 2010	Randomized, controlled	Music group	PD patients *N* = 11, 5/6	67.0 ± 8.1	Walking while listening to music, regular activity	39 30 min, 3/week	Pretraining and post training	Walking with music, walking without music DT: walking with music + subtraction task; walking without music + subtraction, obstacle negotiation trials with music, obstacle negotiation trials without music	Gait speed, cadence ↑*, stride time ↓*	No significant change
		Control group	PD patients *N* = 11, 6/5	64.1 ± 4.2	Regular activity				No significant change	
Dorfman et al., 2014	/	/	Elderly idiopathic fallers *N* = 10, 7/3	78.1 ± 5.81	DT: treadmill training + phoneme monitoring/arithmetic task/verbal fluency tasks	18 Training time increased gradually, 17–47 min, 3/week	Pretraining, post training, and 1-month follow-up	Walking, 6-MWT, TUG, dynamic gait index (DGI), BBS, frontal assessment battery, verbal fluency, TMT, subtraction DT: walking + subtraction task	Gait speed, step length, subtraction task ↑*	After training, gait speed, step length, BBS, DGI, subtraction task ↑*, time to TMTb, stride time variability ↓*
Fraser et al., 2014	/	Dancing and pelvic floor training	Mixed urinary incontinence (MUI) and executive function (EF) deficits women *N* = 23, /	70.4 ± 3.6	Static pelvic floor muscle training (20 min, 5/week), video game dancing	12 60 min, 1/week	Pretraining and post training	Stroop task, TMT, 2-back WM, walking DT: walking + 2-back WM	DTC to 2-back errors ↓*	RT of Stroop task & TMT ↓*, accuracy of Stroop task, TMT score ↑*
Gassner et al., 2022	Randomized	Treadmill group	PD patients *N* = 49, 12/37	60.5 ± 9.1	Treadmill walking training,	10 57 min, 5/week	Pretraining and post training	Walking, BBS, MoCA, UPDRS-III, 2-MWT DT: walking + subtraction task	DTC to gait speed ↓*; gait speed, stride length, swing time ↑*, stance time ↓*	Gait speed, stride length, 2-MWT, BBS ↑*, UPDRS-III ↓*
		Physiotherapy group	PD patients *N* = 51, 14/37	61.7 ± 8.1	Physiotherapy					
Ghadiri et al., 2022	Randomized	DT group	Women with dementia *N* = 19	72.75 ± 6.01	Walking/running/hopping + counting/naming	30 50 min, 3/week	Pretraining and post training	ST: walking (12 min) DT: walking + counting	DTC to walking ↓* after training,	Gait speed, stride length ↑*
		Iranian dance group	Women with dementia *N* = 19	73 ± 6.5	Dance exercises					
Killane et al., 2015	/	FOG group	PD patients with FOG *N* = 13, /	64.2 ± 2.4	DT: VR maze game + Stroop test	8 20 min, 4/week	Pretraining and post training	Steeping-in-place, visual oddball task DT: steeping-in-place + visual oddball task	Stepping time, rhythmicity, RT ↓*	Stepping time ↓*
		Non-fog group	PD patients without FOG *N* = 7, /	64.0 ± 1.6					Stepping time ↓*	Stepping time, RT ↓*
Kuo et al., 2022	Single-blinded, randomized, controlled	Cognitive DT training	MCI patients *N* = 9, 8/1	IQR = 80	DT: walking + repeating phrases/counting numbers/phonemic games/having conversation/sentence reciting	24 45 min, 3/week	Pretraining, post training, and 1-month follow-up	Walking, TMT, digit span MDT: walking + carrying a glass of water CDT: walking + subtraction task	Speed, cadence, stride length ↑*, stride time ↓* in CDT; cadence ↑*, stride time ↓* in CDT; speed, cadence, stride length, stride time ↑*, DTC to gait speed, spatial variability ↓* in CDT	Speed, cadence, stride length ↑*, stride time ↓*
		Motor DT group	MCI patients *N* = 11, 10/1	IQR = 78	Walking + holding balls/rising umbrella/waving rattle/beating a castanet/bouncing a basketball					Speed, cadence, stride length ↑*, spatial variability, stride time ↓*; time for TMTb ↓*
		Physiotherapy group	MCI patients *N* = 10, 5/5	IQR = 79	Muscle stretching/balance/gait training				No significant change	No significant change
Lemke et al., 2019	Double-blinded, randomized, controlled	DT group	Patients with dementia *N* = 56, 39/17	82.7 ± 6.2	10-m walking DT: walking + arithmetic task	20 90 min, 2/week	Pretraining, post training, and 3-month follow-up	Walking, strength task, 2-forward calculation, 3-backward calculation DT: walking + calculation task/verbal fluency, strength + verbal fluency	DTC to gait speed, step length ↓* in walking + 2-forward calculation; DTC to gait speed, cadence, step length ↓*, response rate, strength ↑* in walking + 3-backward calculation	3-Backward calculation ↑* after and follow-up
		Control group	Patients with dementia *N* = 49, 37/12	82.6 ± 5.8	Low-intensity strength training, flexibility exercises				No significant change	No significant change
Liao et al., 2019	Single-blinded, randomized, controlled	VR-cognitive-motor DT group	MCI patients *N* = 18, 11/7	75.5 ± 5.2	VR tasks training: taking mass rapid transit, kitchen chef, convenience store clerk, Tai Chi, football	36 60 min, 3/week	Pretraining and post training	TMT, SCWT, walking DT: walking + subtraction task/carrying a glass of water	Gait speed, stride length ↑* in walking + subtraction; DTC to cadence ↓* in walking +subtraction; gait speed, stride length ↑* in walking + water carrying	Gait speed, stride length, SCWT ↑*, time to TMTb ↓*
		Traditional cognitive-motor DT group	MCI patients *N* = 16, 12/4	73.1 ± 6.8	Standing, walking, reaching, turning, and rising from a chair DT: walking + reciting poems/naming task/mathematic task				Gait speed, stride length, cadence ↑* in walking + subtraction	Gait speed, cadence, SCWT ↑*, time to TMT(b)↓*
Meester et al., 2019	Single-blinded, randomized, controlled	Cognitive-motor DT group	Stroke patients *N* = 26, 11/15	60.85 ± 14.86	DT: walking + subtraction/clock face task/verbal fluency/listening/planning…	20 30 min, 2/week	Pretraining, post training, and 22-week follow-up	Walking (2 min), MoCA, Barthel ADL Index DT: walking with distraction	Walking distance ↑* after and follow-up Cognitive responses ↑*	Walking distance ↑*
		Control ST group	Stroke patients *N* = 24, 13/11	62.25 ± 15.53	Walking				Walking distance ↑*	Walking distance ↑*
Pang et al., 2018	Single-blinded, randomized, controlled	DT group	Stroke patients *N* = 28, 6/22	59.9 ± 6.8	DT training (standing/ walking + naming/ remember/ counting), flexibility exercises (stretching)	24 60 min, 3/week	Pretraining, post training, and 8-week follow-up	Forward walking, TUG, obstacle crossing test., verbal fluency, subtraction task DT: forward walking/TUG/obstacle crossing + verbal/subtraction task	DTC to time ↓* in TUG + verbal task, forward walking + subtraction/verbal task	No significant change
		ST group	Stroke patients *N* = 28, 8/20	61.2 ± 6.2	Standing, walking				No significant change	
		Control group	Stroke patients *N* = 28, 10/18	62.4 ± 6.3	Upper-limb exercise					
San Martin Valenzuela et al., 2020	Single-blinded, randomized, controlled	DT group	PD patients *N* = 23, 12/11	66.38 ± 7.06	DT: walking + verbal fluency/auditory recognition/visual recognition/motor tasks	20 60 min, 2/week	Pretraining, post training, and 8-week follow-up	Frontal assessment battery, TMT, walking DT: walking + watching clock/talking/listening/motor task	Velocity, stride length ↑* after and follow-up; the velocity is statically faster than ST group	Velocity, stride length, cadence ↑* after and follow-up; the velocity is statically faster than ST group
		ST group	PD patients *N* = 17, 5/12	64.75 ± 8.77	Walking training				Velocity, stride length ↑* after and follow-up	Cadence ↑* follow-up, TMTa ↑* after training
Strouwen et al., 2019	Randomized	Consecutive task training	PD patients *N* = 65, /	66.05 ± 9.3	Gait practice, cognitive exercise (verbal fluency, decision making, working memory, mental tracking)	24 30 min, 4/week	Pretraining, post training, and 12-week follow-up	DT: walking + backward digit span task/auditory Stroop task/typing task	Gait speed ↑*	/
		Integrated DT training	PD patients *N* = 56, /	65.80 ± 9.19	DT: gait training + cognitive exercise					
UzunkulaoGlu et al., 2020	Double-blinded, randomized, controlled	ST balance training group	Osteoarthritis patients *N* = 25, 16/9	73.6 ± 5.6	Balance training: tandem standing, semi-tandem stand, single/double leg standing, numbers of stopping	12 45 min, 3/week	Pretraining and post training	BBS, TUG, walking (10 min), static and dynamic scores (kinesthetic ability trainer), activities-specific balance confidence (ABC) DT: TUG/walking + backward counting/days counting	TUG, gait speed ↑*	TUG, gait speed, BBS, static and dynamic scores, ABC scale ↑*; number of stopping ↓*
		DT balance training group	Osteoarthritis patients *N* = 25, 18/7	72.3 ± 5.5	Balance training DT: balance training + song singing/backward counting/days counting					
Wollesen et al., 2021	/	DT group	PD patients *N* = 17, 3/14	70.1 ± 7.4	ST: movement endurance, strength training DT: walking + talking, walking + instruction follow	4 60 min, 4/week	Pretraining and post training	ST: normal walking, fast walking DT: walking + visual-verbal Stroop test	Gait speed, step length ↑*	Normal walking speed, step length ↑*
Yang et al., 2019	Single-blinded, randomized, controlled	Cognitive-motor DT group	PD patients *N* = 6, 2/4	65.0 (presented as the median)	DT: walking + repeat words/subtraction task/count number backward/talk/singing	12 30 min, 3/week	Pretraining and post training	Walking, TUG, fall efficacy scale (FES) Cognitive DT: walking + subtraction task Motor DT: walking + tray carrying	Double support time ↓*; stride length ↑* in CDT; stride time variability ↑* in MDT	Gait speed, stride length ↑*, double support time ↓*, TUG performance ↑*
		Motor DT group	PD patients *N* = 6, 2/4	69.5	DT: walking + ball holding/bouncing				Stride time variability ↓* in MDT	No significant change
		Control group	PD patients *N* = 6, 2/4	66.5	Gait training				No significant change	No significant change

PD, Parkinson disease; FOG, freezing of gait; 6-MWT, 6-min walk test; TUG, timed-up-and-go; MoCA, Montreal Cognitive Assessment; BBS, Berg Balance Scale; SCWT, Stroop Color and Word Test; TMT, Trail Making Test; FSST, Four Square Step Test; RT, reaction times; SPPB, short physical performance battery; VR, virtual reality; UPDRS, Unified Parkinson’s Disease Rating Scale; IQR, interquartile range; /, no report. The symbol “/” represents no report;

*p < 0.05.

#### Combined tDCS and training intervention

Transcranial direct current stimulation (TDCS) was delivered during task training, and different quantities of training sessions (1 vs. 9 vs. 36) were applied in these combined methods’ studies. Particularly, one study applied the same duration of exercise training and tDCS (Schneider et al., 2021), while longer exercise durations were applied and tDCSs were delivered for the first 20 min in the other studies (Schabrun et al., 2016; Liao et al., 2021) (Table 4).

**TABLE 4:** Summary of the tDCS and training parameters and procedure used in combination and task performance.

References	Study design	Participants (*N*, female/ male)	Age (mean ± SD)	Stimulation & training protocol										Training task	Assessment tasks	Outcome	
				Polarity	Electrodes size (cm)	Area	Montage (anodal and cathodal)		Intensity (mA)	Duration (min)	Ramp-up (s)	Stimulation sessions	Training sessions			DT performance	ST performance
Liao et al., 2021	Double-blinded, randomized, sham-controlled	MCI patients *N* = 10, 8/2	72.6 ± 4.1	atDCS	5 × 7	L-DLPFC	F3	The right supraorbital region	2	20	20	36 20 min, 3/week	36 40 min, 3/week	Tai Chi (40 min every session, tDCS was delivered the first 20 min)	Walking, MoCA, visual WM, Tower of London Task, Trail Making Test, Stroop Color and Word Test, Chinese Version of Verbal Learning Test DT: walking + subtraction task, walking + water carrying	DTC to gait speed ↓* in walking + subtraction task	No significant change
		MCI patients *N* = 10, 5/5	73.1 ± 4.6	Sham						0.5						No significant change	No significant change
Schabrun et al., 2016	Double-blinded, randomized, sham-controlled	PD patients *N* = 8, 0/8	72 ± 4.9	atDCS	5 × 7	L-M1	C3	The right supraorbital region	2	20	10	9 20 min, 3/week	9 60 min, 3/week	Walking combined with real-life activities tasks training (e.g., listening, speaking, conversing, list recall, and generation)	Walking, TUG, serial reaction time task (SRTT), bradykinesia (hand closing and opening, elbow flexion, hand closing and opening, elbow extension), Trail-making A and B test DT: walking + word list, walking + counting, walking + conversation, TUG + counting, TUG + words	Gait speed, cadence, step length ↑* in walking + counting/ word/ conversation; double support time ↓* in walking + word/ conversation; TUG speed ↑*; accuracy ↑* in TUG + count/ word	TUG speed ↑*; right arm movement speed ↑*
		PD patients *N* = 8, 6/2	63 ± 11.0	Sham						0.3						Gait speed, cadence, step length ↑* in walking + counting/ word/ conversation; double support time ↓* in walking + word/ conversation; TUG speed ↑*	TUG speed ↑*; right arm movement speed ↑*
Schneider et al., 2021	Cross-over, double-blinded, randomized, sham-controlled	HE *N* = 25, 20/5	73.9 ± 5.2	HD-tDCS	π	M1 + L-DLPFC	F3, Cz	AF4, CP1, FC1, FC5	3	20	59	1 20 min	1 20 min	Walking on a VR treadmill	Walking, standing (30 s), subtraction task, SCWT, Symbol Digit Modalities Test (SDMT) DT: walking + subtraction task, Standing + subtraction task	DTC to gait speed, stride time, step regularity, stride time variability, swing time variability ↓*	The accuracy of SCWT ↑*
														Seated		No significant change	No significant change
				Acti-sham			FC1, Cz	CP1, F3, AF4, FC5	0.5					Walking on a VR treadmill		No significant change	No significant change

HE, healthy elderly; PD, Parkinson’s disease; MCI, mild cognitive impairment; RT, reaction times; WM, working memory; SCWT, Stroop Color and Word Test; MoCA, Montreal Cognitive Assessment; TUG, timed-up-and-go.

*p < 0.05.

### Study outcomes

In the included studies, the primary outcomes were dual task cost (DTC) and gait-related parameters in ST and DT conditions, such as gait speed, cadence, and step length. DTC refers to the percentage change in performance markers from single to dual task (Manor et al., 2016), the performance outcome could be time, dimensions, speed, and accuracy among others.

$$D\,T\,C=\left(\frac{D\,T\,p\,e\,r\,f\,o\,r\,m\,a\,n\,c\,e-S\,T\,p\,e\,r\,f\,o\,r\,m\,a\,n\,c\,e}{S\,T\,p\,e\,r\,f\,o\,r\,m\,a\,n\,c\,e}\right)\times 100$$

The secondary outcomes were spatial-temporal parameters. Single motor tasks were assessed by time, speed, cadence, step length, center of pressure, or sway area. Single cognitive tasks were assessed via accuracy, error rate, reaction time, or scores.

### Single session tES on dual-task performance

#### In healthy elderly

Ljubisavljevic et al. (2019) reported that there was no significant change in the DT walking performance after one session of 2 mA anodal tDCS over the L-DLPFC in the healthy elderly. Manor et al. (2016), nevertheless, reported a significantly lower DTC to standing, walking, and a lower error rate in the healthy elderly following anodal tDCS over the L-DLPFC compared to sham stimulation. Findings from Sayig-Keren et al. (2023) also supported that DTC to walking was decreased after one session either anodal tDCS over L-DLPFC or 6 HZ tACS over L-FPN. Significant decreased DTC during standing, assessed by sway speed and sway area following the intervention targeting only L-DLPFC and both L-DLPFC and the primary somatosensory cortex (SM1), was also observed (Zhou et al., 2021). But this was not observed for the acti-sham and SM1 only interventions. In addition, a similar observation was made for DTC during walking, as measured through speed (Table 1). A noteworthy observation for the studies that found enhanced DT performance is a simultaneous absence of alterations in ST performance following the intervention.

#### In geriatric patients

Only two studies explored the effects of anodal tDCS over the left DLPFC on DT performance in old PD patients. Swank et al. (2016) reported there was no significant enhancement in DT standing after one 20-min tDCS intervention with F3-F4 montage. However, Mishra and Thrasher, 2021 reported increased dual gait speed in the real tDCS condition while using anodal tDCS with the F3-FP2 montage but the stimulation was delivered for 30 min.

### Task training on dual-task performance

#### In healthy elderly

A few studies reported limited effects of task training on DT performance in the healthy elderly. In a study conducted by Hiyamizu et al. (2012), participants were randomly allocated into a balance group and a balance-cognitive group which involved calculation, visual search, and verbal fluency tasks training. After 24 × 60-min training sessions over 12 weeks, no significant changes in DT performance during concurrent balance standing and Stroop task were observed in either group. A similar finding was reported when 12 sessions (gradually increased from 18 to 30 min in 5 weeks) of virtual reality (VR) force plate-based DT training (Delbroek et al., 2017), or 4 × 45-min balance/gait training sessions combined with working memory/calculation task over 4 weeks were applied in the healthy elderly (Plummer-D’Amato et al., 2012).

However, increased number of studies reported significantly improved DT performance in healthy old adults after motor-cognitive DT training was applied (Bherer et al., 2005, 2008, 2021; Fraser et al., 2016; Gregory et al., 2016; Heinzel et al., 2017; Brustio et al., 2018; Bagheri et al., 2021; Tasvuran et al., 2021), such as faster gait speed (Rezola-Pardo et al., 2019) and faster initiation time of steps (Pichierri et al., 2012a) after 24 × 60-min concurrent balance and cognitive training sessions over 12 weeks, as well as lower step time variability after 52 × 60-min concurrent walking and memory training sessions over 26 weeks (Eggenberger et al., 2015). Wollesen et al. (2017b) applied both single walking and DT walking (concurrent walking and attention distraction task/visio-spatial executive task) for 12 × 60-min over 12 weeks in the healthy old adults. Compared to baseline, increased step length was observed after training, which was in line with their previous study (Wollesen et al., 2015).

The effects of ST training on DT performance were also explored. Nevertheless, it is contentious whether ST training has the same role in improving DT performance in the healthy elderly. Silsupadol et al. (2009) divided the participants into balance training group and DT training group, in which old participants were asked to perform standing/walking and objects naming or remembering tasks simultaneously. It was found that after 12 × 45-min training sessions over 4 weeks, balance performance and DT gait speed in the DT training group increased significantly, while no significant change was observed in the balance group. Recent studies reported that standing/walking training for 12 × 60-min sessions over 6 weeks (Tasvuran et al., 2021) did not alter DT performance in the healthy elderly. A similar finding was observed after healthy participants received strength/balance training for 24 × 30-min sessions over 12 weeks (Hiyamizu et al., 2012) or 4 × 45-min sessions over 4 weeks (Plummer-D’Amato et al., 2012). Nevertheless, some studies reported that fitness training for 18 × 60-min sessions over 3 weeks (Agmon et al., 2015), balance training for 24 × 30-min sessions over 12 weeks (Reve and de Bruin, 2014) or 18 × 60-min over 6 weeks (Javadpour et al., 2022), 36 × 30-min bicycle riding sessions over 12 weeks (Raichlen et al., 2020), 20 × 60-min Karate training sessions over 10 weeks (Pliske et al., 2016), or 52 × 30-min Tai Chi training sessions over 26 weeks (Wayne et al., 2015), contributed to enhancing DT ability in healthy old subjects (Table 2).

#### In geriatric patients

Based on the data so far, both ST and DT Training have been shown to have noticeable effects in improving DT ability in old PD patients (Chang et al., 2020; D’Cruz et al., 2020; Gassner et al., 2022; Table 3).

In the first study using ST training to improve DT ability in geriatric patients (de Bruin et al., 2010), PD patients in the training group were asked to perform single tasks, consisting of walking and regular activity, whereas participants in the control group conducted only regular activity. After 39 × 30-min training sessions over 13 weeks, patients in the training group showed increased gait speed, cadence, and lower stride time during walking combined with performing subtraction tasks, while no significant change was observed in the control group. Strouwen et al. (2019) applied gait training and cognitive auditory training in isolation in PD patients for 24 × 30-min training sessions over 6 weeks, significant faster gait speed during concurrent walking and cognitive tasks (digit span task, auditory Stroop task, and typing task) were observed. UzunkulaoGlu et al. (2020) reported that after 12 × 45-min training sessions over 4 weeks, gait speed under DT conditions improved in osteoarthritis patients participating in both the single-balance training group (e.g., single/double leg standing) and the dual-balance training group (single balance combined with counting/singing). This finding was supported by Conradsson and Halvarsson (2019). A similar single-balance and dual-balance training protocol was implemented in female participants with osteoporosis, but for 36 × 45-min training sessions over 12 weeks. In both groups, gait speed, cadence, and step width improved under DT conditions.

The effects of DT training on PD patients have been further explored. Yang et al. (2019) randomly allocated PD patients into (1) cognitive-motor DT group, in which participants were asked to conduct walking and a subtraction task simultaneously, or (2) motor DT group, where participants were asked to walk while holding a ball, or (3) control group, where patients only performed gait training. Shorter double support time and longer stride length were observed in the motor-cognitive DT group after 12 × 30-min sessions over 4 weeks, while the motor DT group showed significantly reduced stride time variability during DT walking. No improvement was observed in the control group. A study conducted by Killane et al. (2015) applied concurrent VR maze game and Stroop test in PD patients with freezing of gait (FOG) and without FOG separately. After 8 × 20-min sessions over 2 weeks, significantly shorter stepping time in concurrent steeping and visual oddball tasks has been observed in both groups. San Martin Valenzuela et al. (2020) allocated 40 PD patients into a cognitive-motor DT group and a walking group. Participants in the DT group were asked to walk while carrying out one cognitive task such as verbal fluency, or auditory recognition task while those in the ST group performed only walking training. After 20 × 60-min sessions over 10 weeks, the DT gait speed and stride length of patients increased significantly in both groups, notably, the DT group exhibited an even greater increase in gait speed compared to the single walking group.

Many clinical studies support the efficiency of task training on improving DT ability in elderly patients including stroke (Pang et al., 2018; Meester et al., 2019), MCI (Liao et al., 2019; Kuo et al., 2022), osteoporosis (Conradsson and Halvarsson, 2019), osteoarthritis (UzunkulaoGlu et al., 2020), idiopathic fallers (Dorfman et al., 2014), and executive functioning disorders (Fraser et al., 2014). However, two included studies which allocated dementia patients and inconsistent results were reported. Chen and Pei (2018) applied music DT training, where patients were asked to perform concurrent singing/instrument playing and walking tasks for 8 × 60-min training sessions over 8 weeks. No enhancement was observed within concurrent walking and forward/backward digit recall tasks but better cognitive function, assessed by a single trail making test. Another study reported enhanced cognitive function and better performance under concurrent walking and calculation task after concurrent walking and arithmetic training were applied for 20 × 90-min training sessions over 10 weeks (Lemke et al., 2019).

### Combined tDCS with task training

As previous studies reported positive results for tDCS and task training in improving DT capacity, only several studies combined these two approaches and explored its effectiveness on DT performance in older adults (Table 4).

#### In healthy elderly

To our knowledge, only one study explored its effectiveness in healthy elderly. In this study, participants received either real HD-tDCS at 3 mA for 20 min, or sham stimulation (0.5 mA, 20 min, near zero normal electric fields) targeting the left M1 and L-DLPFC while walking on a VR treadmill, or real HD-tDCS while sitting (Schneider et al., 2021). Compared to the sham condition, lower DTC during concurrent walking/standing and subtraction tasks was observed after real stimulation.

#### In geriatric patients

In a study conducted by Schabrun et al. (2016), 16 PD patients were recruited and received 9 sessions of either 20-min anodal tDCS over left M1 at 2 mA or sham tDCS paired with 60-min motor-cognitive DT training, which entailed walking combined with real-life activities such as talking. The sessions took place 3 times per week and tDCS was delivered for the first 20 min of each session. Following the intervention, both groups showed a significant enhancement in DT walking, including increased gait speed, cadence, step length, and reduced double support time under walking while conversing/performing a word-list task. Moreover, significantly increased gait speed and lower error rate under dual Time-Up-and-Go test were observed.

Recently, Liao et al. (2021) applied 36 sessions of combined either 20 min 2 mA anodal tDCS over the L-DLPFC or sham stimulation with 40 min Tai-Chi training over 12 weeks in old MCI patients. TDCS was delivered for the first 20 min. Compared to sham stimulation, the anodal tDCS group showed significantly improved walking performance during concurrent walking and subtraction/water carrying tasks after intervention.

## Discussion

### Does single session tDCS influence DT ability in the elderly?

In the above-mentioned studies, L-DLPFC was the main target area as it is highly related to executive function (Zhou et al., 2014). A combined tDCS and functional near-infrared spectroscopy study revealed lower oxygenated hemoglobin response in the left prefrontal cortex within DT condition after 2 mA, 20 min tDCS over L-DLPFC was applied, suggesting that L-DLPFC tDCS modulates the prefrontal recruiting, and the reduction of DTC may be due to the reduced oxygen consumption (Jor’dan et al., 2022) as neuroimaging study which mentioned above reported a higher activation in the healthy elderly during DT condition. Compared to tDCS over the right DLPFC or sham stimulation, anodal tDCS over the L-DLPFC can significantly enhance executive functions within conflict-related tasks (Dubreuil-Vall et al., 2019). Patel et al. (2019) noted that the improvement in cognitive and behavioral skills induced after tDCS over M1 could be attributed to cortical plasticity, potentially triggered by a decrease in gamma-aminobutyric acid concentration, which has an important role during motor learning. The higher performance after tDCS over DLPFC may be due to the increased cortical excitability in the executive control and ventral attention networks within the brain (Soleimani et al., 2022).

Nevertheless, the low number of studies and inconsistent findings within these studies make the ability of a single tDCS session to adequately enhance DT performance in the healthy elderly or geriatric patients inconclusive. As shown in Table 1, Manor et al. (2016) reported significantly lower DTC to standing and walking in DT condition after 2 mA anodal tDCS with F3-FP2 montage for 20 min. However, results from Ljubisavljevic et al. (2019) suggested that 30 min anodal tDCS with F3-FP2 montage at 1.5 mA had no effects on DT ability. This inconsistence is less likely due to the study design as most studies applied double-blinded, crossover design. However, multiple other factors may contribute to this discrepancy, such as sample size, stimulation parameters (intensity, duration, electrodes sizes), as well as the participants’ characteristics [exercise frequency, or the proficiency in skills (Furuya et al., 2014)]. Therefore, more studies are needed to explore the effects of tES on DT performance in the healthy elderly.

Similarly, there is insufficient evidence to suggest that tDCS has a facilitative effect on DT performance in PD patients. Mishra and Thrasher (2021) reported enhanced DT walking after 1.5 mA, 20 min anodal tDCS over L-DLPFC, while Swank et al. (2016) reported no significant changes but a trend of reduced DTC on walking was observed after anodal tDCS via two electrodes of size π (3.14 cm^2^). The result can be attributed to various factors, e.g., the disease stages of patients. Mishra recruited participants with mild-moderate severity (stage I–III, assessed by the Hoehn and Yahr scale), while Swank included patients with stage II. In addition, stimulation parameters also contributed to the inconsistent results. For instance, a 30-min stimulation duration was utilized in the study conducted by Mishra, whereas Swank applied tDCS for 20 min. Mishra and Thrasher (2021) positioned the anodal electrode over F3, with the cathode electrode placed over the right supraorbital region, while a F3-F4 montage was applied by Swank et al. (2016). The inconsistent size of the electrodes (35 cm^2^ vs. 3.14 cm^2^) also contributed, as Hashemirad et al. (2017) reported that tDCS with small electrodes (3 cm^2^) over left M1 or L-DLPFC did not affect cognitive functions.

Interestingly, only one study reported improved ST (component tasks) performance after intervention (Mishra and Thrasher, 2021; Table 1). Out of these six studies, most reported enhanced DT performance without altering single cognitive or motor task performance. For instance, Manor et al. (2016) reported notable DT standing/walking performance after a single session of tDCS, assessed by a slower sway velocity/increased gait speed and subtraction error rate within DT condition, but no significant improvements in walking, standing, or subtraction task was observed when these tasks were performed in isolation after intervention. Ceiling effects such as low error rate for cognitive tasks and relatively high motor performance in the baseline within ST condition may be an underlying confounding factor since healthy old participants were recruited, or the functional integrity of the underlying network was at its limits and could not be further improved by the stimulation (Ljubisavljevic et al., 2019; Zhou et al., 2021). This could be explored further in the following studies.

Based on the limited research, it is insufficient to demonstrate the effectiveness of HD-tDCS with multi-area stimulation in improving DT performance in the elderly. Therefore, to explore further the possibilities of utilizing anodal tDCS and HD-tDCS of the L-DLPFC in promoting DT capacity in the elderly, more studies should be conducted in the future.

### Does task training influence DT ability in the elderly?

The existing evidence suggests that the DT capacity of the elderly can be enhanced by training, especially, cognitive-motor DT training. In comparison to tDCS studies, almost all training studies applied multiple sessions. Recent research reported enhanced DT gait speed and DT cognitive performance after one walking training session with the split-belt treadmill in both PD patients and healthy old adults, with greater improvements obtained while two belts were set at different speeds (D’Cruz et al., 2020).

The underlying mechanism between improved DT performance and task training has not been addressed, but a few hypotheses have been proposed. Some theorists suggested that DTC might originate from two separate sources: (1) incomplete conversion of verbal descriptions to procedural memory (i.e., muscular memory) and (2) conservative execution control with postponing certain stages of a task while another task is in progress due to the response-selection bottleneck (Pashler, 1994; Schumacher et al., 2001).

In the former context, DT ability can be regarded as one particular skill. According to the skilled performance model, every single skill is the programmatic knowledge in the form of condition-action rules. This programmatic knowledge can be converted from declarative knowledge through practice, and once the conversion is completed, performing skill/actions in an easy way (performing DT at a lower cost) could be possible (Schumacher et al., 2001). Schumacher et al. (2001) applied concurrent cognitive-demanding tasks and visual-auditory tasks in young adults, participants were asked to respond to the visual stimulus (e.g., circle) by pressing keyboards with one hand, and report numbers (e.g., 1, 2, and 3) orally for the auditory stimulus simultaneously. The training lasted for 8 sessions, participants performed single tasks in session 1, while both STs and DT were performed from sessions 2 to 8. Results have shown that the DTC to reaction times was significantly lower after practice, and even a “perfect time-sharing” was observed between two tasks within DT condition in session 8. That could partially explain why significant improvements were observed in both ST and DT conditions in studies which utilized identical single and dual tasks during training and assessment phases. For instance, Heinzel et al. (2017) combined visual working memory (WM) task training and auditory WM task training simultaneously for 12 × 45-min sessions over 4 weeks in the healthy elderly, participants showed notable lower DTC to WM and a significantly higher accuracy within the same DT condition, as well as better single WM performance during assessment phase. Gregory et al. (2017) applied a concurrent walking and arithmetic task in the training phase for 78 × 40-min sessions over 24 weeks, increased gait speed and step length were observed in both DT walking (concurrent walking and calculation task) and single walking condition.

The second theory holds that the response-selection stage of the second component task is processed only when the stage of the first task has been completed (Strobach et al., 2013). This delay is considered to be the source of DTC. Evidence provided by Strobach et al. (2013) suggested that the reason for higher DT performance after training/practice might come from speed-up central response-selection stages of both tasks. In this study, eight training sessions similar to Schumacher et al. (2001) were applied but followed up by two more sessions. The old visual stimulus was intermixed with novel visual stimulus (different shape, e.g., triangle), and participants were instructed to respond to the stimulus with the unpracticed hand in session 10. Results revealed a great reduction of reaction times in both component tasks during the ST condition, and significantly reduced reaction times within the DT condition, which was maintained in the last two sessions, suggesting that practice can facilitate such response-selection processing as well. Besides, this gained facilitation can be still observed in the new DT condition.

To address why practice effects were maintained in new conditions, “transfer effects” and “task coordination skills” were cited. The acquisition of the task coordination skill is related to the optimization of executive function within DT condition through training, and this optimization is associated with the enhancement of DT, as DT is regarded as a particular part of the execution function (Kramer et al., 1995; Strobach et al., 2014). A previous study showed that task coordination skills can be acquired only in DT conditions and the acquired task coordination skills are independent of the training settings and tasks (Kramer et al., 1995). Namely, these skills are transferable and can benefit new, unpracticed conditions or DTs, which have (1) identical or similar structures, such as motor-cognitive DT, motor-motor DT, or cognitive-cognitive DT, or (2) contain the same component task, for instance, practiced walking task and a changed subtraction task, practiced subtraction task and a different walking task, or (3) contain the same/similar input or output, for example, practiced/similar auditory/visual input, practiced/similar keyboard response. As a consequence, even though the DTC cannot be eliminated, extensive practice-gained task coordination skills induce an optimization of executive function, which enables efficient processing of two concurrent task streams and reduces the cost of performing dual tasks. Moreover, the “transfer effects” partially explained why significant improvements can still be observed after training in studies that applied different DT conditions in the training phase and assessment phase, e.g., whole body stretch exercise combined with visual discrimination task for training but concurrent walking and n-back task for assessment (Fraser et al., 2016), or concurrent balance and cognitive task for training but simultaneous walking and backward counting task for assessment (Rezola-Pardo et al., 2019).

However, improved DT performance was observed in studies, that applied STs for training in the healthy elderly (Wollesen et al., 2017a; Raichlen et al., 2020), PD patients (Chang et al., 2020; Gassner et al., 2022), stroke patients (Meester et al., 2019), and women with osteoporosis (Conradsson and Halvarsson, 2019). Pashler and Baylis (1991) suggested that the practice effects produced by ST training might shorten the response selection stage during the DT condition because, as discussed above, the enhancement of DT performance after ST training is not related to task coordination skills. Strobach et al. (2013) found that ST training might shorten other processing stages, such as the initial perception stage, or final motor stage since the ST used for training in these studies was one component of DT used for assessment (Strobach et al., 2013). Namely, the transfer effects might be observed when the same task exists both in the training phase and as one component of the DT during the assessment phase, e.g., calculation task and aerobic task were used for training in isolation but performed concurrent walking and calculation task in DT assessment (Chang et al., 2020), or walking task and standing task were practiced in isolation and combined walking and backward counting task was applied during DT assessment (Javadpour et al., 2022), or balance and postural training were applied in the training phase and concurrent standing and tone discrimination task was applied for the assessment (Agmon et al., 2015).

One interesting observation was that DT performance also improved in studies that did not have the same component task during the training and evaluation phases. For instance, Raichlen et al. (2020) applied only bicycle riding training for 36 × 30-min sessions over 4 weeks in healthy old adults, an increased DT cognitive function, assessed by significantly higher accuracy during a concurrent walking and subtraction task, was observed after training. Reve and de Bruin (2014) applied single strength training, balance training, as well as cognitive training for 24 × 30-min sessions over 12 weeks in the healthy elderly, lower DTC to walking was observed in a concurrent walking and object naming task. Furthermore, a greater improvement in cognitive function was also observed within ST condition after training, assessed by enhanced performance on the trail-making test and simple reaction time task. In these cases, it seems that the boosted DT performance was not related to the transfer effects, task coordination skills, or shortened certain stages in DT condition.

A few studies applied VR-based training (Delbroek et al., 2017) or game-based training (Pichierri et al., 2012b; Fraser et al., 2014), and positive effects were observed in old patients (Killane et al., 2015) as well as healthy elderly (Bisson et al., 2007; Eggenberger et al., 2015; Bagheri et al., 2021). Game-based training is widely used nowadays and its effects on brain plasticity were explored, such as improving cognitive function in old adults (Maillot et al., 2012), and decreasing depression in young individuals (Li et al., 2022). In the future, these methods could be popular technologies in improving DT capacity.

### Does the combined approach influence DT ability in the elderly?

Transcranial direct current stimulation combined with proper task training can be a promising tool for improving DT capacity in the elderly. However, further studies are required to validate its effectiveness.

The study conducted by Liao et al. (2021) emphasized the role of tDCS, as DT performance was significantly improved in patients with MCI only after combined Tai Chi training with anodal tDCS, but not sham, over DLPFC.

Schabrun et al. (2016) demonstrated that training played a key role in the improvement of DT performance as both DT training conjunct with 2 mA 20 min anodal tDCS over M1 and sham showed significantly improved DT performance.

The study which was conducted by Schneider et al. (2021) highlighted the function of combination. In this study, improved DT walking performance and cognitive ST performance were observed after participants received concurrent HD-tDCS over M1 and DLPFC and walking training. However, neither the HD-tDCS condition nor concurrent walking and sham stimulation condition showed improved ST or DT performance in healthy elderly after one session intervention. Contrarily, a study by D’Cruz et al. (2020) reported the effectiveness of single-session split-belt treadmill walking training in DT performance enhancement. This may indicate that more complicated training tasks can yield the same or more pronounced training effects with less training effort than training with simple tasks. In this context, split-belt training trained the motor coordination of the lower and upper limbs in addition to walking training when compared with conventional treadmill walking training.

We suggest the combined approach can be a valid tool to improve DT performance, however, the effects of independent components need to be further explored. For example, the role of training and tES in the enhancement of DT performance if exists, and whether tDCS accelerates the training process when compared with pure training. Furthermore, more evidence is required from aged healthy people and geriatric patients.

### Limitations and future direction

This review is a traditional, systematic, qualitative clinical review of studies involving some form of tDCS and treatment techniques. It must be pointed out that some training-only studies were not RCTs. Further limitations were the variability in the number of sessions and type of tasks for the different studies involving task training as well as the low sample size of multiple studies.

In the future, a more in-depth exploration of the montage of tES intervention should be conducted, despite specific positions or cortices, brain networks such as the frontoparietal network can also be targeted. Additionally, it is important to highlight that all included tES-only studies in this review utilized only a single intervention session, further investigation through multi-session interventions is recommended as it has been reported that repeated tES can induce long-term potential effects and boost cognitive enhancement and lasting up to 1 month (Korai et al., 2021; Antonenko et al., 2023). In the realm of training studies or clinical rehabilitations, virtual reality techniques could be an optimized tool moving forward. Besides, the understanding of the underlying mechanisms behind the improved DT performance can be facilitated while a comprehensive, multimodal method is adopted in the assessment, for example, EEG-fMRI.

## Conclusion

This review is the first article to discuss the relationship between different potentially therapeutic approaches – tES, training, and DT performance in old adults. Sixty-four studies including tDCS only, task training only, and the combination of both to improve DT capacity in both healthy elderly and old patients were discussed in this study. This study provides an overview that task training, particularly cognitive-motor DT training, can be a validated method for enhancing DT performance in the elderly. The effectiveness and potential mechanisms of task training in improving DT abilities in older adults were also further addressed. However, the possibility of tDCS-only intervention in improving DT capability in older adults requires further exploration. The potential of tACS, the combination of tES and training in the enhancement of DT performance in the elderly deserves further investigation.

## Data availability statement

The original contributions presented in this study are included in this article/supplementary material, further inquiries can be directed to the corresponding author.

## Author contributions

YJ: Conceptualization, Data curation, Formal analysis, Investigation, Methodology, Project administration, Visualization, Writing – original draft. PR: Validation, Visualization, Writing – review & editing. AA: Supervision, Validation, Writing – review & editing.
